# Exploring the biological basis of fatigue using primary Sjögren's syndrome as a disease model

**DOI:** 10.1186/1471-2474-14-S1-A1

**Published:** 2013-02-14

**Authors:** Andini Savietri Natasari, Jessica Tarn, Roman Fischer, Dennis Lendrem, Wan Fai Ng

**Affiliations:** 1 Musculoskeletal Research Group, Institute of Cellular Medicine, Newcastle University, Newcastle Upon Tyne, NE2 4HH, UK; 2 Henry Wellcome Building for Cellular and Molecular Physiology, Roosevelt Drive, Oxford, OX3 7BN, UK

## Background

Despite being a common debilitating symptom in many chronic conditions such as autoimmune diseases or cancers, the pathophysiology of fatigue is still unclear. Unsurprisingly, there is no effective treatment for sufferers [1]. Using primary Sjögren’s Syndrome (pSS) as a disease model, this study aimed to determine biomarkers of fatigue and to better understand its pathophysiology.

## Methods

Serum samples from pSS patients with different fatigue levels and healthy controls were analysed using cytometric bead array (CBA) and mass spectrometry-based label-free quantitative proteomics. The CBA, proteomic and clinical data were statistically analysed for their relationship with fatigue.

## Results

Statistical analysis concluded that fatigue was negatively correlated with IP10, IL12p70, ESR and IgG, but positively correlated with platelets and C4 (p-value <0.05, Benjamini-Hochberg correction). Moreover, most protein levels were significantly higher in patients compared to healthy controls (p-value <0.05). An observed pattern was apparent that protein levels of patients with high fatigue lie between healthy controls and low fatigue patients. Additionally, Multiple linear regression analysis identified low IgG, high platelets and low IFNα as key predictive factors for fatigue. Furthermore, after quantitation and identification, the proteomic analysis confirmed 9 proteins to be significantly down-regulated and 3 proteins to be significantly up-regulated (p-value <0.05) in patients with high fatigue compared to those with low fatigue. The results also indicated that there was influence of confounders in the analysis, suggested by different symptoms being significantly correlated with similar proteins.

## Discussion

Based on the observations, it is indicated that insufficient levels of serum inflammation markers are features of pSS patients suffering from fatigue.

## Conclusion

Using different approaches, we have identified 19 potential biomarkers for fatigue that is hoped to add our understanding of fatigue.
